# Phylogenetic association of Cyprinid (Schizothorax) inferred from complete mitochondrial genome

**DOI:** 10.1080/23802359.2020.1827996

**Published:** 2020-11-06

**Authors:** Aqsa Rehman, Muhammad Fiaz Khan, Saira Bibi, Faisal Nouroz

**Affiliations:** a Department of Zoology, Hazara University, Mansehra, Pakistan; b Department of Bioinformatics, Hazara University, Mansehra, Pakistan

**Keywords:** Phylogenetic association, snow trout, mitochondrial DNA

## Abstract

Whole mitochondrial DNA (mtDNA) of *S.esocinus*and *S. plagiostomus* was found to be 16,591 and 16,564 bp respectively with 13 protein-coding genes, 2 rRNAs genes, 22 tRNA genes and 2 non-coding region. For valuing the phylogenetic relationship of a species on the basis of whole mitogenome are considered to be a great importance. In this study, we sequenced whole mitogenome of *S.esocinusand S. plagiostomus* and compared with the whole mitogenome sequences of members of other families (Balitoridae, Nemanchillidae and Cobatidae). The monophyly of the family Cyprinidae and a clade comprising Balitoridae and Nemanchillidae while Cobatidae with paraphyletic origin was strongly supported by the resultant phylogenies, recognized that Cyprinidae was closely related with the family Cobatidae than other ones. The results indicated that whole mitochondrial genome has a great importance in studying variation in genes and phylogenetic relationship in the subfamily Schizothoracinae. This data offering the molecular phylogenetic frame work of important *Schizothorax* species found in Swat.

The Subfamily Schizothoracinae, belongs to family Cyprinidae and lived in the area and countries around the Qinghai-Tibet Plateau (Chen and Cao 2000) including China, India, Nepal, Bangladesh, Burma, Kazakhstan, Kyrgyzstan, Tajikistan, Pakistan, Afghanistan, Turkey, Iran, etc. Schizothoracinae includes about 12 genera and more than 100 species, of which 76 species or subspecies from 11 genera are distributed in China (Mirza 1991; Chen et al. 2000). The rivers, snow and streams of Jammu and Kashmir of India, is inhabited by the Schizothoracinae fish, which are commonly named as snow trout (Durham *et al*. 2002). In order to reveal the taxonomy and phylogenetic of Schizothoracinae, a series of pronounced developments have been made, but these studies have also produced some problems such as, one argument was the monophyletic origin of Schizothoracinae. The monophyly of Schizothoracinae strengthened by the datasets analyses, consisting of all morphological characteristics, mitochondrial gene sequences and single nuclear genes (He et al. 2004; Qi et al. 2007). For resolving phylogenetic trees branches, it is extensively known that information attained from a single gene is often inadequate (Sinclair et al. 2002; Afonso *et al*. 2011). Mitochondrial DNA have genetic information providing a base to accomplish and keep the biological diversity and for the explanation of evolutionary accounts of varied biological species it allows the researchers (Bernt et al. 2013). The mtDNA is one of the most suitable marker for phylogenetic studies (Avise 2012). An effective example is shown (Saitoh et al. 2006), where phylogenetic relationship of major Cypriniformes lineages were resolved conspicuously. One major concern related to the use of mtDNA in resolving the organism phylogeny is that the whole mtDNA is fundamentally a single locus and the linkage of all mitochondrial genes might increase systematic errors e.g. compositional biases (Gadagkar et al. 2005). Phylogenetic and evolutionary relationships between diverse fish fauna and genome have played a major role (Karaiskou et al. 2003; Domingues et al. 2007). On the basis of genes, analysis of phylogenetic relationship of Cyprinid taxa helpful in understanding the functional divergence and speciation (Kong et al. 2007). The present study, we find out the phylogenetic relationship of *Schizothorax* species with that of the other fishes of order cypriniformes on the basis of whole mitogenome.

Present study was carried out in District Swat, located in Khyber Pakhtunkhwa, a mountainous part of Pakistan comprises of various valleys on the hills and alpine meadows on the boundaries, latitude between 34°30′00″–35°50′00″N and 72°05′00″–72°50′00″E, district is located with an altitudinal range from 500 to 6,500 m above the sea level with a surface area of 5,037 km^2.^ (GOP 1999). Fish samples of two *Schizothorax* species were collected out from River Swat with the help of cast nets. Voucher specimens (SP10FND), (SE2FNE) were fixed in 10% formalin and transported to Department of Zoology Hazara University Mansehra, Pakistan museum Muscles samples were stored in 95% ethanol.

The standard high salt extraction method was used for the amplification of the mitochondrial DNA of *Schizothorax* species (Miller et al. 1988). Polymerase chain reaction (PCR) were carried out for amplification and sixteen sets of primers were intended which were based on original mitochondrial genomes DNA sequences of cyprinid fish. Cocktail reaction included The 25 µL to 6 µL of 10 ×buffer, every nucleotide of 1.5 µL (dNTP), every primer of 1 µL, Taq DNA polymerase about 1.5 unit, template DNA of 1–2 µL. The process of thermocycling was started for 5 min at 94 °C, followed through 20 cycles at 94 °C for 30 s, 56 °C for 50 s, and 72 °C for 1 min 30 s, with 0.1 °C reducing the temperature for annealing to every cycle, with the annealing temperature at 54 °C Then 12 other cycles used to, there was an end of final cycle at 8 min extension. On 1.2% of Agarose gel in 1 × Trisacetate-EDTA buffer for all samples, PCR product was electrophoresed about 1 µl at 80 V for 30 min then staining with ethidium bromide, as well as in the Gel-Doc system visualized under Ultraviolet illumination. For sequencing, these PCR products were sent to the Sangon biotech company (Sangon Biotech Company Shanghai).

The DNA sequences were aligned by using Clustal W software (Takamatsu et al. 1998) and BioEdit (Hall 1999). The phylogenetic association was inferred by MEGA 6.0 (Tamura et al. 2013). To find out the phylogenetic relationships, the mtDNA of *Schizothoraxesocinus* was compared with the sequences of other cyprinids and members of other families of order cypriniformes which were retrieved from NCBI. The MEGA 6.0 was used to construct the phylogenetic trees and also find their nucleotide composition. All the gaps and missing data were eliminated. The phylogenetic tree was constructed based on mitochondrial genome sequences of *S. esocinus* and *S. plagiostomus* with other species to confirm the taxonomy and phylogenetic relationships.

## Results

**Figure 1.**
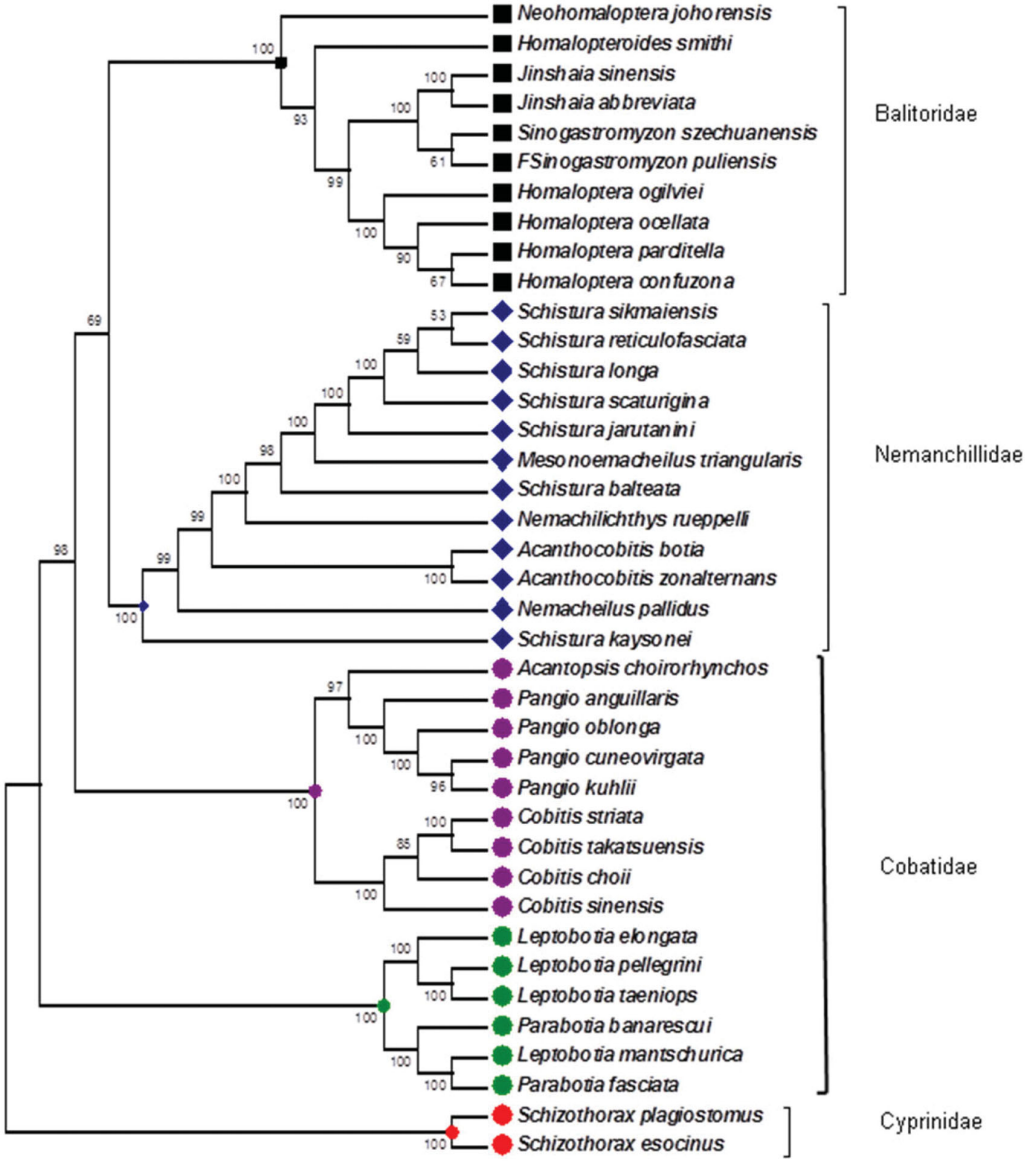
Neighbor-joining phylogenetic tree of Schizothoracinae with other families of Order cypriniformes on the basis of complete mitogenome.

Phylogenetic tree divided the various sequences into two different lineages. Each lineage was comprised of different genus which were further divided into subsequent species. These species formed groups representing different families Balitoridae, Nemanchillidae, Cobatidae and family Cyprinidae. In which *Schizothoraxplagiostomus (KT184924)* and *Schizothoraxesocinus (KT21088)*, clustered on the single branch and showing the close homology with one another and with the members of family Cobatidae (*Leptobitia* and *Parabotia)* supported by the 100% bootstrapped values. It is clear from the tree that Cobatidae have a paraphyletic origin as Cobitis, Acanthopsis, Pangio formed one group while Leptobia and Parabotia formed another group. Cyprinids formed monophyletic group. Balitoridae was also paraphyletic because of the uneven positions of *Neohomalopterajohorensis* while family Nemanchillidae formed a monophyletic group consisted of genus such as *Acanthocobitis, Nemachilichthys, Schistura, Mesonoemacheils* and *Nemacheilus* which was supported by high bootstraps values as indicated in the tree (Figure 1).

## Discussion

In present study we compared Schizothoracinaefishes (*S. esocinus* and *S. plagiostomus)* with other fishes of order cypriniformes on the basis of whole mitogenome. We find out their phylogenetic relationships and also homology in their MtDNA. The complete mitochondrial genomes of the *S. esocinus* and *S. plagiostomus* species were found to be 16,591 and 16,564 bp respectively, consisted of 13 protein coding genes, 2 rRNAs genes, 22 tRNA genes and 2 non-coding region. The relationships among the clades derived from the data of the whole mitogenome sets of four families of order Cypriniformes was created. It was cleared from tree that Cyprinidae was closely related with the family Cobatidae than other ones. Our results differ from the earlier hypotheses proposed by (Wu et al. 1981; Siebert 1987) assembled on morphological characters who suggested that the family Cyprinidae was more closely related to the Balitoridae than to another family Cobitidae and family Cyprinidae was monophyletic while Cobatidae was paraphyletic in origin. These results were supported by the findings of (Saitoh et al. 2006).

Our study revealed less genetic variation in *S. plagiostomus* and *S. esocinus* due to some reasons (1) at genomic level, they showed the slow rate of evolution (2) and their population history. The less variation in the mitochondrial DNA sequences among the species of *Schizothorax* may be due to the imperfect organization in lineages (Qi et al. 2007). Yang et al. (2012) described that mitochondrial DNA sequences were incompetent to differentiate some species in *Schizothorax*on morphological characteristics. In the subfamilies of Cyprinidae, phylogenetic interrelationships have been widely studied from anatomical, morphological and molecular insights (Cavender 1992; Zardoya and Doadrio 1999).

## Conclusion

The study of phylogenetic relationship helps to investigate the taxanomy of schizothoracinae and their relation with other species and provide a step for further investigation of phylogeny of fishes.

## Data Availability

The data that support the findings of this study are openly available in repository under Accession: KT184924.1https://www.ncbi.nlm.nih.gov/nuccore/ KT210882) GI: 926459750 in gene bank.
